# AKR1C2 silencing promotes ferroptosis and inhibits proliferation, migration, and invasion in lung cancer cells

**DOI:** 10.1371/journal.pone.0325995

**Published:** 2025-06-18

**Authors:** Yi Cui, Zhihui Liu

**Affiliations:** 1 The First Clinical Medical School of Guangzhou University of Chinese Medicine, Guangzhou, People’s Republic of China; 2 Department of Clinical Laboratory, The First Affiliated Hospital of Guangzhou University of Chinese Medicine, Guangzhou, People’s Republic of China; 3 Shenshan Hospital, The First Affiliated Hospital of Guangzhou University of Chinese Medicine, Shanwei, People’s Republic of China; The First Affiliated Hospital of Nanjing Medical University, CHINA

## Abstract

**Background:**

Lung cancer is the leading cause of cancer-related deaths worldwide, so research and development of potential therapeutic targets is urgent.

**Methods:**

The target gene, AKR1C2, was screened using the TCGA and FerrDb databases. The expression of AKR1C2 in lung cancer, its correlation with the clinical characteristics of patients, and the biological roles and molecular mechanisms involved were assessed by bioinformatics. The expression levels of AKR1C2 in normal lung epithelial cells and lung cancer cells were compared by qRT-PCR and Western blot. The effects of AKR1C2 knockdown on the malignant phenotype of lung cancer cells were evaluated using Transwell, wound healing, and CCK8 assays. Finally, the impact of silencing AKR1C2 on malondialdehyde, reactive oxygen species, Fe^2+^ levels, and ferroptosis-related genes in lung cancer cells were experimentally investigated.

**Results:**

AKR1C2 is highly expressed in lung adenocarcinoma tissues, and the expression level correlates with patient gender, tumor stage, lymph node metastasis, and prognosis. AKR1C2 is associated with substance metabolism, steroid hormone biosynthesis, cellular lipid metabolism, and oxidoreductase activity. AKR1C2 had high expression levels in A549 cells. AKR1C2 silencing inhibited cell viability, invasion, and migration. In A549 cells, AKR1C2 knockdown markedly raised the levels of reactive oxygen species, malondialdehyde, and Fe²⁺. The knockdown of AKR1C2 regulated the expression of ferroptosis-related genes.

**Conclusion:**

Our study demonstrates that AKR1C2 knockdown promotes ferroptosis and inhibits malignant biological behaviors in lung cancer cells.

## 1. Introduction

Globally, lung cancer stands as a prominent cause of cancer-associated mortality [1]. Of these, lung adenocarcinoma (LUAD) is the most common histological type of lung cancer [2]. Over the past decade, significant advancements have been achieved in the treatment of lung cancer, particularly through the application of molecularly targeted therapies and immunotherapies. Despite these progressions, the 5-year survival rate for patients remains below 30%, with metastasis and drug-resistant recurrence persisting as two major unresolved challenges [3]. Studying the underlying molecular mechanisms and creating more potent, molecularly targeted treatment approaches are therefore imperative.

Ferroptosis is a new programmed cell death pathway first discovered and proposed by Dixon et al [4] in 2012, which is uniquely biochemically characterised by aberrant lipid peroxidation, reactive oxygen species (ROS) accumulation, and substantial elevation of iron levels [5]. In recent years, Ferroptosis has had a significant regulatory role in lung cancer, according to an increasing number of research findings [6]. MAX’s Next Tango promotes the proliferation of LUAD cells and reduces their sensitivity to chemotherapy by inhibiting the spermine/spermine N1-acetyltransferase 1-associated ferroptosis pathway [7]. Knockdown of myeloma overexpressed genes promotes ferroptosis and inhibits the growth and proliferation of LUAD [8].

A member of the human Aldo-keto reductases (AKRs) family, Aldo-keto reductase family 1 member C2 (AKR1C2), regulates steroid hormones and is aberrantly expressed in many cancers [9]. According to certain research, AKR1C2 expression in lung cancer is strongly associated with resistance to cisplatin and adriamycin [10–12]. However, it has not yet been investigated or documented whether it has a role in controlling LUAD cell migration, invasion, proliferation, and ferroptosis.

Therefore, the aim of this study was to characterize AKR1C2 expression levels based on the LUAD dataset in TCGA database and to identify the functional roles of AKR1C2 and its relationship with ferroptosis by bioinformatics and cell experiments.

## 2. Materials and methods

### 2.1. Target gene screening

The dataset of LUAD was obtained from TCGA database, and significant differentially expressed genes between cancerous and normal tissues in this dataset were screened by DESeq2 package analysis of R software. The screening condition was set as |log2FoldChange| > 2, *P* adjust <0.05. The differential genes obtained were intersected with ferroptosis-related genes downloaded from the FerrDb database to obtain the differential genes related to ferroptosis in lung adenocarcinomas, and volcano diagrams were plotted using the ggplot2 package. Subsequently, the target gene of this study were identified after a literature search.

### 2.2. Correlation analysis between AKR1C2 and patients’ clinical characteristics and prognosis

Relevant clinical information was downloaded from the TCGA database and integrated with the AKR1C2 expression data to analyze the expression levels of the target gene in different patient ages, genders, tumor grades, and TNM stages to assess the relationship between AKR1C2 and the clinical characteristics of the patients.

The relevant survival information was downloaded from the TCGA database and integrated with the AKR1C2 expression data, in which the patients were divided into two groups of high and low expression based on the expression level of the target gene. Subsequently, the survival curve was plotted by the survival package in R to determine the effect of high and low expression of AKR1C2 on the prognosis of the patients.

### 2.3. Functional and pathway analysis of AKR1C2

GO, KEGG, GSEA enrichment analysis of target genes was performed by using R’s org.Hs.es.db package and clusterProfiler package, and the results were visualized by drawing the corresponding histograms using the ggplot2 package.

### 2.4. Cell culture

The team of Academician Lin Lizhu from the Oncology Department at the First Affiliated Hospital of Guangzhou University of Chinese Medicine generously donated human normal lung epithelial cells (BEAS-2B). Meanwhile, LUAD cells (A549) were acquired from Zishan Biotech (Wuhan, China). Both cell lines were grown in RPMI 1640 medium (Gibco, USA) with 10 μL/ml of penicillin/streptomycin (Gibco, USA) and 10% fetal bovine serum (FBS) (Procell, China) added. The culturing was conducted at a temperature of 37°C with 5% CO_2_.

### 2.5. Transfection

Sangon Biotech (Shanghai, China) provided the small interfering RNA (siRNA), including negative control (si-NC) and si-AKR1C2. A 6-well plate was injected with 3.5 × 10^5^ cells per well, and after 24 h of incubation, when the cell fusion efficiency reached 70–80%. Following the instructions, transfection complexes comprising si-NC or si-AKR1C2 were made using RPMI 1640 medium and Lipo8000TM transfection reagent (Beyotime, China). The cells were then collected for additional research 48 h after transfection.

### 2.6. Quantitative real-time PCR (qRT-PCR)

Trizol reagent (Thermo Fisher Scientific, USA) was used to extract RNA from each set of treated cells. Evo M-MLV RT Premix for qPCR (Accurate Biology, China) was then used to transform this RNA into cDNA. Subsequently, qRT-PCR was performed on the extracted cDNA using a QuantStudio 5 real-time PCR machine (Applied Biosystems, USA), employing the SYBR® Green Premix Pro Taq HS qPCR Kit (Accurate Biology, China). The 2^-ΔΔCT^ approach was used to determine the relative expression levels of the gene’s mRNA. Table 1 lists the precise sequences of the primers used in this investigation, which were obtained from Sangon Biotech (Shanghai, China).

**Table 1:** Primers for qRT-PCR in this study.

Gene	Sequence from 5’-3’
AKR1C2-F	AAGTAAAGCTCTAGAGGCCGT
AKR1C2-R	CTCTGGTCGATGGGAATTGCT
β-actin-F	CTACGTCGCCCTGGACTTCGAGC
β-actin-R	GATGGAGCCGCCGATCCACACGG
SLC7A11-F	GGTACTGCAATCACAATGCCAGA
SLC7A11-R	GCACATGCATCAAGAGTTTCCATAA
GPX4-F	AATTCGCAGCCAAGGACATC
GPX4-R	AGGCCAGGATTCGTAAACCA
ACSL4-F	TGGAAGTCCATATCGCTCTGT
ACSL4-R	TTGGATACAGCATGGTCAAA
FTH1-F	CCCCCATTTGTGTGACTTCAT
FTH1-R	GCCCGAGGCTTAGCTTTCATT

### 2.7. Western blot

RIPA buffer (Beyotime, China) was used to lyse cells, and an Enhanced BCA Protein Assay Kit (Beyotime, China) was used to measure the protein content. Following their separation using 10% SDS-PAGE (Beyotime, China), the proteins were transferred onto 0.45 μm PVDF membranes. These membranes were blocked with Western Blot Blocking Buffer (Beyotime, China) for 15 min at room temperature. Following this, they were incubated overnight at 4°C with primary antibodies against AKR1C2 (1:800, DF3757, Affinity, China), glutathione peroxidase 4 (GPX4, 1:7500, ET1706–45, HUABIO, China), SLC7A11 (1:1500, YP-Ab-17133, UpingBio, China), ACSL4 (1:1500, YP-Ab-12589, UpingBio, China), ferritin heavy chain 1 (FTH1, 1:750, WL05360, Wanleibio, China), and β-actin (1:7500, AF7018, Affinity, China). After that, the membrane was washed and incubated with the second antibody for an hour at room temperature. Lastly, an ultrasensitive ECL chemiluminescent substrate (Biosharp, China) was used to visualize the blot.

### 2.8. Cell counting kit‑8 (CCK‑8)

A549 cells were plated into 96-well plates at a cellular density of 5 × 10^3^ cells per well and then pre-cultured for 24 hours in an incubator. Following this, the cells were transfected based on the experimental grouping. Post-transfection, after a duration of 48 h, 10 μL of CCK-8 reagent (Beyotime, China) was dispensed into each well. The cells were then further incubated at 37°C for an extended period of 1.5 h. The viability of the cell was assessed by using a microplate reader (Thermo Fisher Scientific, USA) at 450 nm and 600 nm.

### 2.9. Transwell assay

Matrigel (Corning, Inc., USA) was first lined in 8 μmpore transwell chambers (LABSELECT, China). Medium containing 10% FBS was placed in the lower chamber, whereas 2 × 10^5^ transfected cells and media devoid of FBS were placed in the top chamber. The cells were fixed for 40 min with 4% paraformaldehyde and stained for 20 min with 0.1% crystal violet solution following a 24-hour culture period. Then the invasive ability of the cells was measured by photographing and counting under an inverted microscope.

The chambers were not coated with Matrigel and were seeded with 1 × 10^5^ transfected cells. The remaining steps were identical to those used in the invasion assay. The migratory capacity of the cells was assessed by photographing and counting the cells under an inverted microscope following fixation and staining.

### 2.10. Wound healing assay

Cells transfected for 48 h in a six-well plate were scratched in parallel with a 200 μL pipette tip, rinsed with PBS, and replaced with the culture that contained 1% FBS. The cell migration at the 0h and 24h scratches were photographed under an inverted microscope.

### 2.11. Detection of intracellular ROS

The treated and collected cells of each group were suspended in DCFH-DA fluorescent probe (Beyotime, China) working solution diluted in FBS-free medium, incubated at 37°C for 20 min without light (mixing upside down every 3–5 min), and washed in FBS-free medium 3 times. A flow cytometer was then used to measure the ROS levels in the cells (BD Biosciences, USA).

### 2.12. Measurement of malondialdehyde (MDA)

The MDA level was evaluated by a lipid oxidation assay kit (Beyotime, China). Cells from individual groups underwent centrifugation for collection, followed by lysis through ultrasonic treatment in an ice-cold bath. After that, they underwent another centrifugation at 12,000 rpm for 10 min at 4°C. After mixing the resulting supernatant with the MDA test reagent, it was heated for 60 min at 100°C in a metal bath. A microplate reader (Thermo Fisher Scientific, USA) set at 532 nm was used to measure the MDA content.

### 2.13. Measurement of iron content

The cells of each group were washed with PBS 3 times. After adding 1 μmol/L of FerroOrange work solution (Dojindo, Japan), the mixture was incubated for 30 min at 37°C. Direct observation and photography of the cells were carried out under an inverted fluorescent microscope (Olympus Corporation, Japan) without washing.

### 2.14. Statistical analysis

GraphPad Prism 9.5 and R 4.3.1 were used for data processing and statistical analysis. To guarantee the precision and dependability of the findings, every experiment was meticulously and independently conducted at least three times. The mean ± standard deviation (Mean ± SD) is used to display all data. We used the Student’s *t*-test method to demonstrate the difference between the two samples. For multi-group comparisons, one-way ANOVA was used. When *P* < 0.05, we considered the observed differences to be statistically significant.

## 3. Results

### 3.1. Identification of AKR1C2 as a target gene and its high expression in LUAD

**Fig 1:**
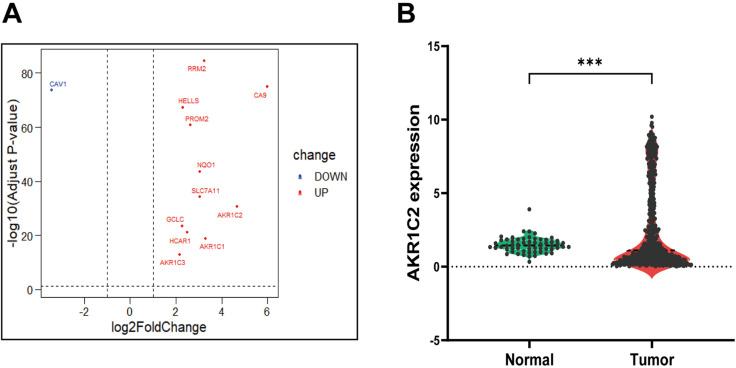
LUAD differential genes associated with ferroptosis.

589 lung cancer cases with RNA sequencing were downloaded from TCGA database, including 533 cancer cases and 56 normal tissue cases. After differential analysis and taking intersections with ferroptosis-related genes, 12 differential genes associated with ferroptosis in LUAD were screened out (11 of which were up-regulated in expression and 1 down-regulated in expression) (Fig 1A). The literature search revealed that no studies have confirmed the correlation between AKR1C2 and ferroptosis in LUAD, whereas the other genes have been studied. So AKR1C2 was identified as the target gene. Meanwhile, the results of differential analysis showed that the expression level of the AKR1C2 gene was significantly higher in tumor tissues compared with normal tissues (Fig 1B).

(A) 12 LUAD differential genes were associated with ferroptosis. 11 of them were up-regulated, and 1 was down-regulated in expression. (B) Expression of AKR1C2 gene in tumor tissues versus normal tissues. AKR1C2 gene expression was significantly elevated in tumor tissues (*P* < 0.001). ***P < 0.001.

### 3.2. Relationship between AKR1C2 expression levels and patients’ clinical characteristics and prognosis

**Fig 2:**
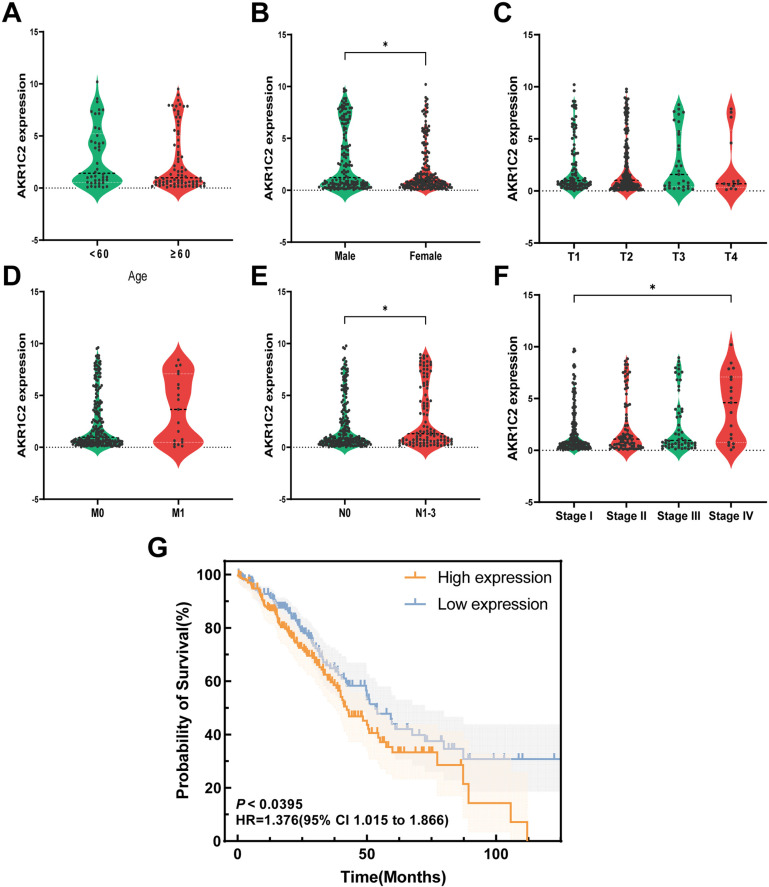
Relationship between AKR1C2 gene expression and clinical characteristics and prognosis of LUAD patients.

Relevant clinical and prognostic information was downloaded from the TCGA database and integrated with the target gene expression data. The correlation between AKR1C2 and clinical characteristics of lung adenocarcinoma patients was predicted, and the results showed that the expression level of AKR1C2 was closely related to gender, tumor stage, and lymph node metastasis of lung adenocarcinoma patients (Fig 2B, E, and F), and was not related to the age of lung adenocarcinoma patients, tumor size, infiltration depth, and distant metastasis (Fig 2A, C, and D). Kaplan-Meier survival curves were also drawn for AKR1C2 expression level and patients’ overall survival time (Fig 2G), from which the survival curves showed that the expression level of AKR1C2 was correlated with patients’ overall survival, and that the overall survival of the group with high AKR1C2 expression was lower than that of the group with low expression, which indicated that the prognosis of the patients with high AKR1C2 expression was worse.

(A) The expression level of AKR1C2 was not related to the age of patients. (B) The expression level of AKR1C2 was related to the gender of patients. The expression level of AKR1C2 in male patients was slightly higher than that in females (*P* < 0.05). (C) The expression level of AKR1C2 was not related to the size and infiltration range of lung cancer. (D) The expression level of AKR1C2 was not related to the distant metastasis of lung cancer. (E) The expression level of AKR1C2 was correlated with lymph node metastasis of lung cancer. The expression level of AKR1C2 in patients with lymph node metastasis was slightly higher than that in those without lymph node metastasis (*P* < 0.05). (F) The expression level of AKR1C2 was correlated with the pathological stage of lung cancer. The expression level of AKR1C2 in patients with pathological stage IV was slightly higher than that in grade I (*P* < 0.05), and the difference between the rest of the grades was not statistically significant. (G) The expression level of AKR1C2 was correlated with the prognosis of the patients. Patients with high expression of AKR1C2 had a worse prognosis (*P* < 0.05). *P < 0.05.

### 3.3. Functional and pathway analysis of AKR1C2

**Fig 3:**
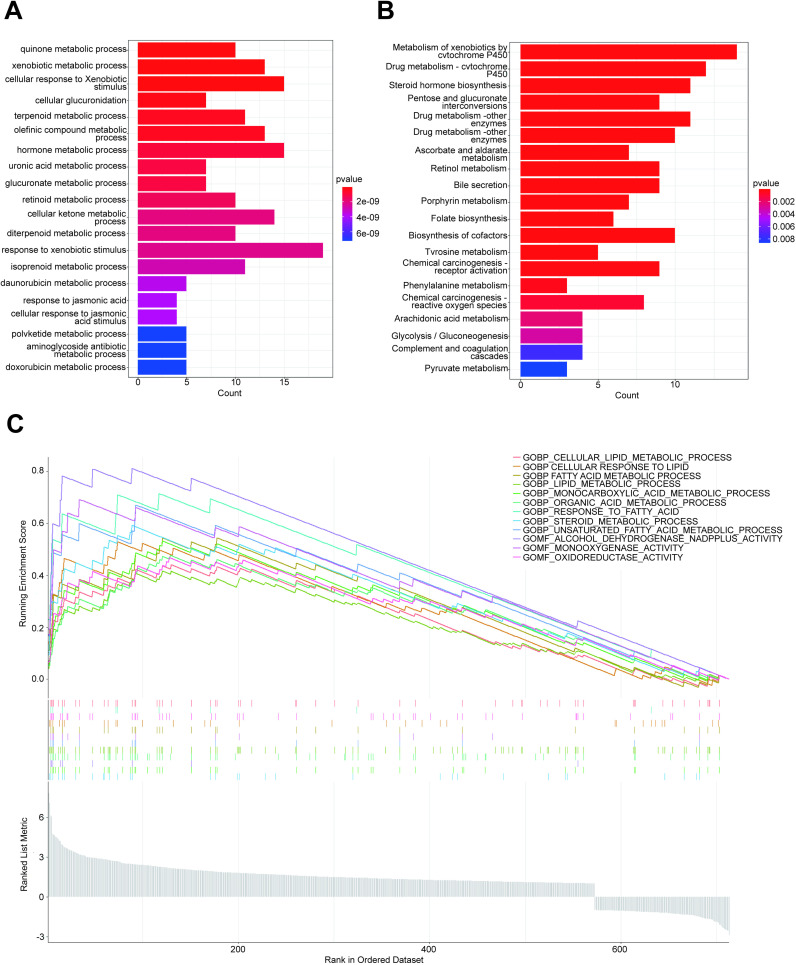
Functional and pathway enrichment analysis of AKR1C2 gene. (A) GO analysis. (B) KEGG analysis. (C) GSEA enrichment analysis.

GO/KEGG analysis showed that the differentially expressed molecular functions of AKR1C2 were mainly enriched in substance metabolism, steroid hormone biosynthesis, and the metabolic processes of doxorubicin and Zoerythromycin (Fig 3A, B). GSEA analysis showed that it was mainly enriched in cellular lipid metabolism, oxidoreductase activity, and other pathways (Fig 3C).

### 3.4. AKR1C2 is highly expressed in A549 cells

**Fig 4:**
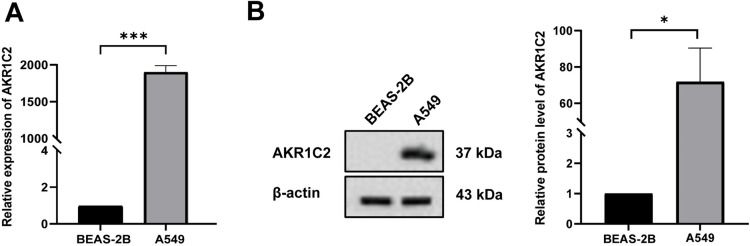
AKR1C2 expression was up-regulated in A549 cells. (A) qRT-PCR was performed to detect the mRNA expression level of AKR1C2 compared to BEAS-2B (n = 6). (B) Western blot detection of the protein expression level of AKR1C2 compared to BEAS-2B (n = 6). ***P < 0.001,*P < 0.05.

**Fig 5:**
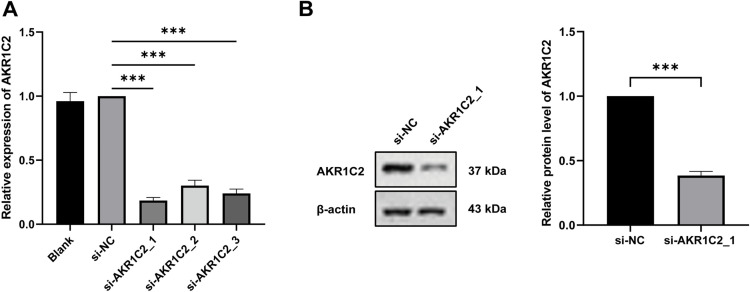
Transfection of A549 cells with siRNA. (A) The knockdown efficiency of AKR1C2 mRNA expression level was detected by qRT-PCR (n = 4). (B) The knockdown effect of AKR1C2 protein levels was detected by Western blot (n = 8). ***P < 0.001. siRNA: short interfering RNA; si-NC: negative control.

We first used qRT-PCR and Western blot to compare the expression of AKR1C2 in normal lung epithelial cells with that in A549 cells in order to gain a better understanding of the function of AKR1C2 in tumor cells. The findings clearly showed that A549 cells’ AKR1C2 expression level was noticeably higher than BEAS-2B cells’ (Fig 4). Next, we knocked down the expression of AKR1C2 to examine its possible impact on the biological behavior of A549 cells. A549 cells transfected with si-AKR1C2_1/2/3 exhibited significant downregulation of AKR1C2 expression. Among these, the si-AKR1C2_1 group demonstrated the most effective knockdown compared to the other two groups (Fig 5). Therefore, si-AKR1C2_1 was selected for subsequent experiments.

### 3.5. Knockdown of AKR1C2 inhibits proliferation, migration, and invasion of A549 cells

**Fig 6:**
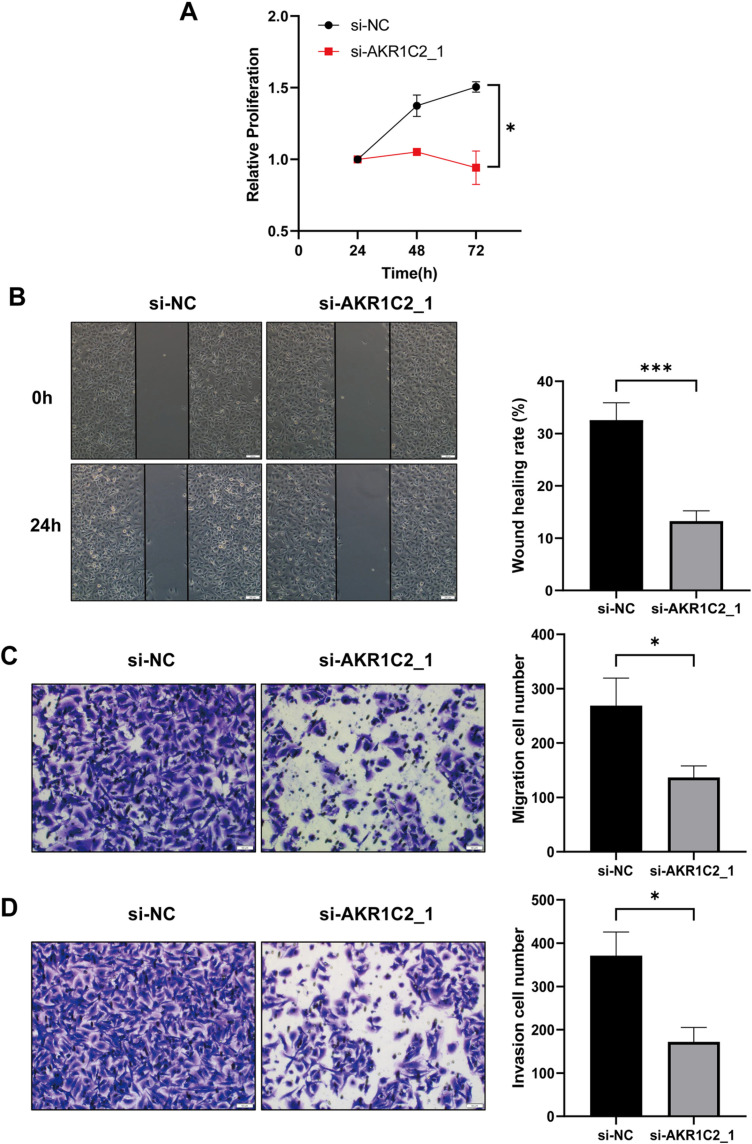
AKR1C2 silencing inhibited A549 cell proliferation, migration, and invasion. (A) CCK8 assay was used to determine the proliferative capacity of transfected cells (n = 6). (B-C) Wound healing (n = 6) and transwell migration assay (n = 5) were used to determine the migratory capacity of transfected cells. (D) Transwell invasion assay was used to determine the invasive capacity of transfected cells (n = 5). *P < 0.05, ***P < 0.001.siRNA: short interfering RNA; si-NC: negative control.

To investigate the role of AKR1C2 in lung cancer cells A549, this study assessed whether AKR1C2 affected the invasion, migration, and proliferation of A549 cells. Firstly, we observed the viability and growth of AKR1C2 knockdown cells by CCK8 cell proliferation assay. The findings demonstrated that AKR1C2 knockdown dramatically decreased A549 cell viability and inhibited cell growth (Fig 6A). According to this, AKR1C2 could be crucial for preserving cell viability as well as encouraging the growth and development of tumors.

Next, we further explored the impact of AKR1C2 knockdown on A549 cell migration by wound healing and Transwell migration. In this experiment, we set up AKR1C2 knockdown and control groups to measure the efficiency of cell migration. The results showed that following AKR1C2 knockdown, the migratory efficiency of A549 cells was markedly decreased (Fig 6B, C), indicating that AKR1C2 is the key to control A549 cell migration.

In addition, Transwell invasion assay was a method to assess the invasive ability of cells. By observing the changes in invasiveness after cell transfection, we found that AKR1C2 knockdown could effectively inhibit the invasive ability of A549 cells (Fig 6D).

Taken together, these data consistently showed that A549 cell invasion, migration, and proliferation were all decreased by AKR1C2 knockdown.

### 3.6. Knockdown of AKR1C2 promotes ferroptosis

**Fig 7:**
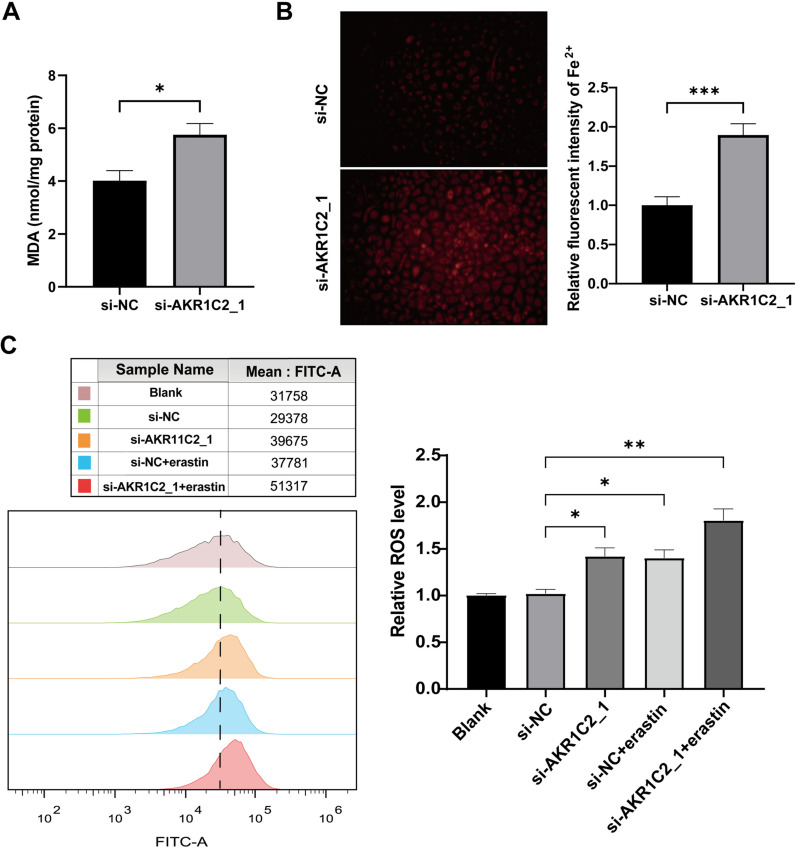
AKR1C2 silencing affects the levels of ferroptosis markers in A549 cells. (A) MDA levels were measured with a kit (n = 6). (B) Fe^2+^ levels were measured with FerroOrange probe (n = 11). (C) DCFH-DA staining was used to estimate intracellular ROS levels by flow cytometry (n = 8). *P < 0.05, **P < 0.01, ***P < 0.001.siRNA: short interfering RNA; si-NC: negative control. erastin: ferroptosis inducer.

**Fig 8:**
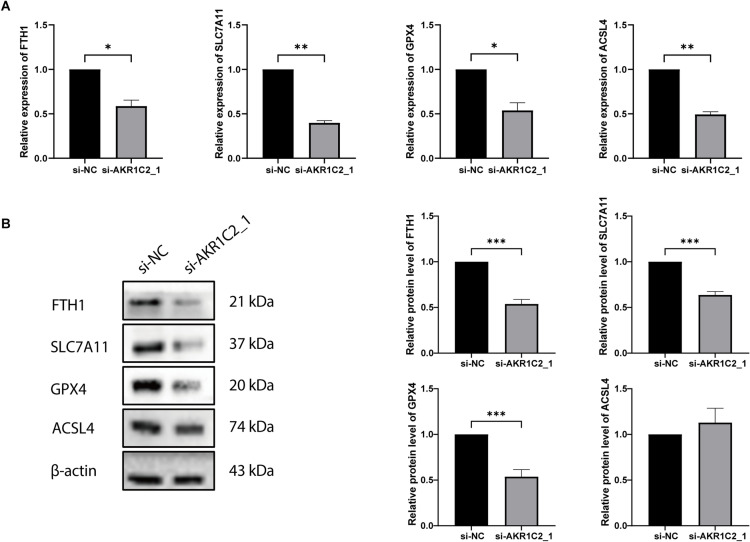
AKR1C2 silencing affected the expression of ferroptosis genes in A549 cells. (A) qRT-PCR to detect the expression of ferroptosis-related genes (n = 3). (B) Western blotting to detect the expression of ferroptosis-related proteins (n = 6). *P < 0.05, **P < 0.01, ***P < 0.001. siRNA: short interfering RNA; si-NC: negative control.

The reduction of AKR1C2 significantly increased MDA and iron content levels (Fig 7A, B). DCFH-DA staining revealed that AKR1C2 downregulation dramatically raised intracellular ROS levels in A549 cells, comparable to the levels observed in cells treated with the ferroptosis inducer erastin. Moreover, AKR1C2 downregulation synergistically enhanced the increase in intracellular ROS levels when combined with erastin treatment in A549 cells (Fig 7C). qRT-PCR (Fig 8A) and Western blotting (Fig 8B) both showed that in AKR1C2-silenced A549 cells, SLC7A11, GPX4, and FTH1 expression were reduced, and the ACSL4 protein level did not significantly change. In conclusion, AKR1C2 knockdown may lead to ferroptosis in A549 cells.

## 4. Discussion

Oncogenes regulate cancer development and progression. With the rapid advancement of precision medicine, novel therapeutic strategies targeting oncogenic pathways have been proposed to improve the prognosis of patients with LUAD [13]. Targeted therapy, either alone or in combination with other modalities, has emerged as a novel therapeutic approach with a potential preventive role in lung cancer [14]. However, the identification of biomarkers for lung cancer remains limited, with only a small proportion of patients currently benefiting from these markers [15]. Therefore, in order to enhance diagnosis and define therapeutic intervention targets, it is imperative to develop more accurate and efficient biomarkers [16].Therefore, in this study, from the perspective of ferroptosis, we screened one gene, AKR1C2, which may be related to ferroptosis and is highly expressed in LUAD tissues through bioinformatics analysis and literature research.

AKR1C2 protein is highly expressed in human liver, stomach, and bladder tissues [9]. It is also aberrantly expressed in various cancers, where it regulates malignant phenotypes and contributes to chemoresistance in tumor cells. Silencing AKR1C2 inhibits triple-negative breast cancer cells’ ability to proliferate, migrate, invade, develop, and spread to the lungs [17]. Inhibition of the AKR1C2 enzyme by ruthenium complexes in chemotherapy-resistant ovarian cancer cell lines has antiproliferative effects [18]. Exosome lncAKR1C2 promotes lymph node metastasis in gastric cancer [19]. Reducing AKR1C2 expression in lung cancer cells increases sensitivity to cisplatin [12]. Meanwhile, the correlation analysis and prognostic analysis in this study showed that the expression level of AKR1C2 was closely related to gender, tumor stage, and lymph node metastasis of LUAD patients and that the prognosis of patients with high AKR1C2 expression was worse. It suggests that AKR1C2 may be involved in regulating the malignant phenotype of LUAD. Consistent with previous studies and bioinformatics analysis, we found that AKR1C2 had a high expression level in A549 cells. Furthermore, we used CCK-8, scratch, and Transwell assays to show that AKR1C2 knockdown prevented A549 cells from growing, proliferating, and migrating.

It has been suggested that AKR1C2 is involved in steroid hormone biosynthesis [20] and ROS formation [21], which can reduce the level of ROS in cancer cells [22], make them resistant to oxidative stress and drug stimulation and ultimately mitigate death [23]. This contributes to the development and progression of malignant tumors. Ferroptosis is essential for controlling the proliferation of tumor cells, which is characterised by elevated iron levels and accumulation of intracellular lipid ROS [5], and induction of ferroptosis has emerged as a possible cancer therapy [24–27]. Furthermore, it has been demonstrated that in melanoma, BET inhibitors enhance GPX4 inhibition-induced ferroptosis through dual downregulation of AKR1C2 [28]. The molecular function and pathway analysis in this study also showed that AKR1C2 is associated with processes such as substance metabolism, steroid hormone biosynthesis and pathways such as cellular lipid metabolism and oxidoreductase activity. Given these findings, AKR1C2 may regulate ferroptosis, making it a possible therapeutic target for LUAD. Investigating its function could aid in identifying potential markers of tumor progression. No study has confirmed the relationship between AKR1C2 and ferroptosis in LUAD cells. In this study, we looked at how AKR1C2 affected markers linked to ferroptosis, and the findings indicated that AKR1C2 knockdown not only downregulated ferroptosis-related genes’ expression levels (FTH1, GPX4, and SLC7A11) but also elevated ROS, MDA, and Fe^2+^ levels. Taken together, we suggest that AKR1C2 may promote LUAD by inhibiting ferroptosis, and AKR1C2 knockdown helps to promote ferroptosis in lung cancer cells, thereby inhibiting lung cancer progression. These findings strongly imply that AKR1C2 targeting may be a viable approach to creating innovative treatment plans specifically designed to fight lung cancer. However, our current findings are limited to investigations in a single LUAD cell line (A549), and we have not yet fully elucidated the molecular mechanisms through which AKR1C2 regulates ferroptosis. Therefore, it is necessary to conduct more experiments to further validate and explore.

## 5. Conclusions

In conclusion, our study indicates that AKR1C2 knockdown promotes ferroptosis in LUAD cells and inhibits their proliferation, migration, invasion, and tumor growth. AKR1C2 may be a potential target for the treatment of lung cancer.

## Supporting information

S1 Raw images All raw western blot images within this study. (PDF)

S1 File Original data. (XLSX)

S2 File RCode used in this study. (DOCX)
