# Supplementary material for: AKR1C2 silencing promotes ferroptosis and inhibits proliferation, migration, and invasion in lung cancer cells
Source: PLoS One. 2025 Jun 18;20(6):e0325995. doi: 10.1371/journal.pone.0325995 (PMC12176218; doi:10.1371/journal.pone.0325995)

Fig4 B. Western blot detection of the protein expression level of AKR1C2 compared to BEAS-2B

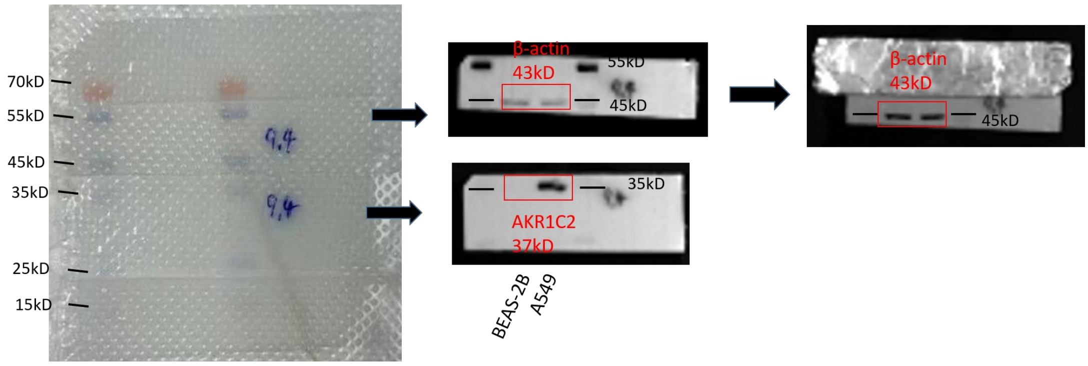

Fig 5 B. The knockdown effect of AKR1C2 protein levels was detected by Western blot

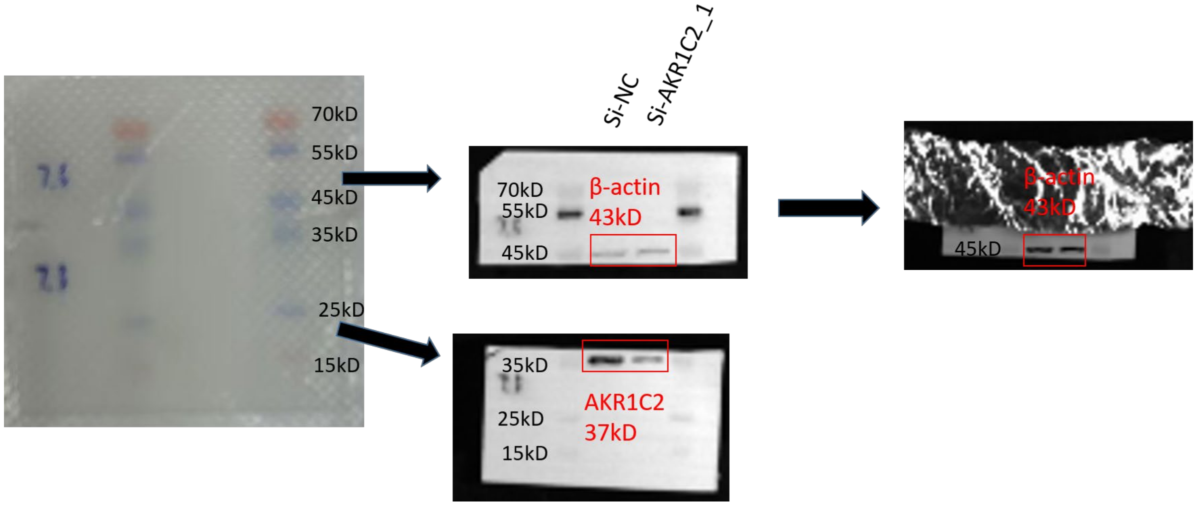

Fig8 B. Western blotting to detect the expression of ferroptosis-related proteins (ACSL4 and GPX4)

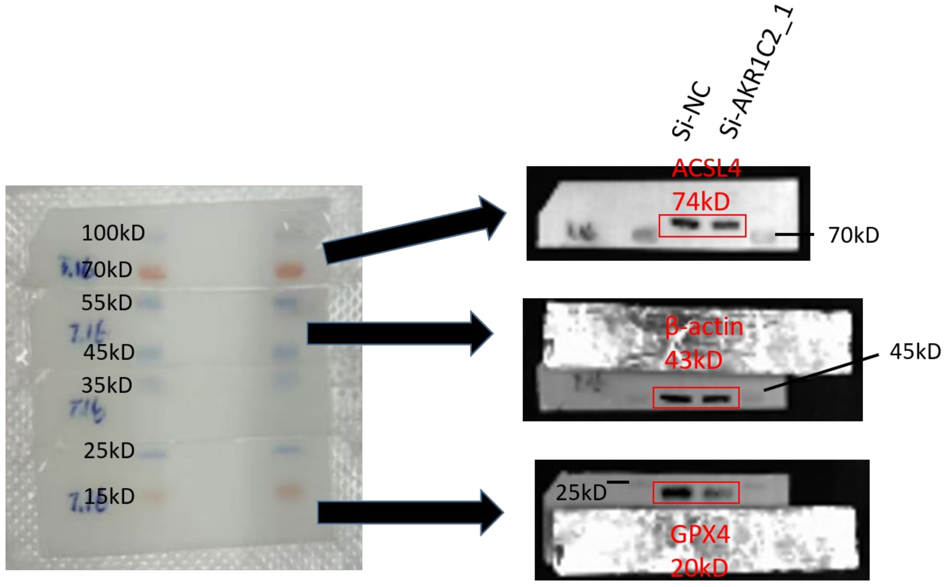

Fig8 B. Western blotting to detect the expression of ferroptosis-related proteins (SLC7A11 and FTH1)

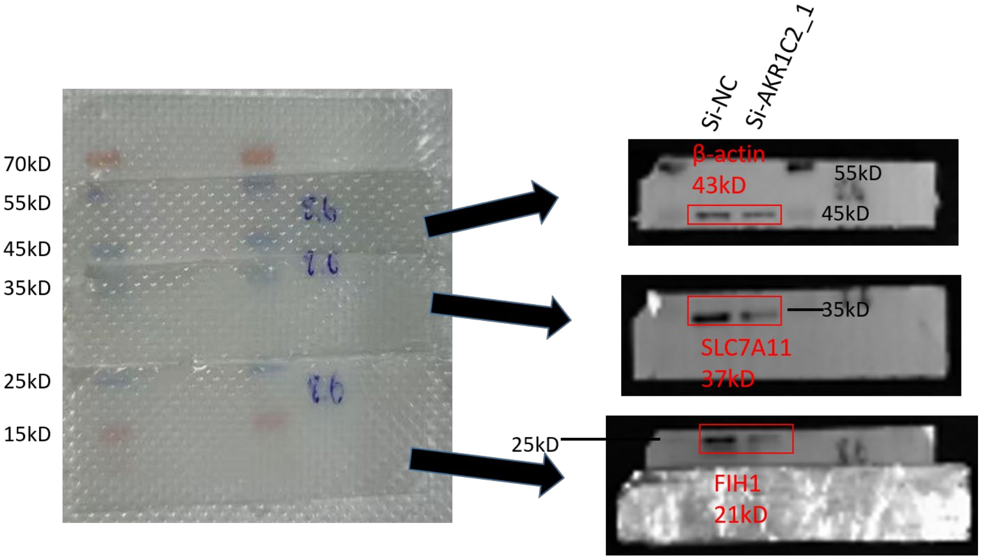

Supplement: S1 Raw images — (PDF) [file pone.0325995.s001.pdf]
