# Supplementary material for: AKR1C2 silencing promotes ferroptosis and inhibits proliferation, migration, and invasion in lung cancer cells
Source: PLoS One. 2025 Jun 18;20(6):e0325995. doi: 10.1371/journal.pone.0325995 (PMC12176218; doi:10.1371/journal.pone.0325995)
Supplement: S2 File — (DOCX) [file pone.0325995.s003.docx]

**Differential Analysis**

setwd("LUAD")

#install.packages("tidyverse")

library(tidyverse)

counts1 = read.table(file = 'TCGA-LUAD.htseq_counts.tsv', sep = '\t', header = TRUE)

rownames(counts1) <- counts1[,1] #Alt <-

counts1 = counts1[,-1]

counts1 <- counts1[,substr(colnames(counts1),14,16)%in% c("01A","11A")]

table(substr(colnames(counts1),14,16))

rownames(counts1) <- substr(rownames(counts1),1,15)

counts <- ceiling(2^(counts1)-1)

write.table(counts,"counts.txt",sep = "\t",row.names = T,col.names = NA,quote = F)

write.csv(counts, file = "counts.csv")

Ginfo_0 <- read.table("gene_length_Table.txt",sep = "\t",check.names = F,stringsAsFactors = F,header = T,row.names = 1)

Ginfo <- Ginfo_0[which(Ginfo_0$genetype == "protein_coding"),]

comgene <- intersect(rownames(counts),rownames(Ginfo))

counts <- counts[comgene,]

class(counts)

class(comgene)

Ginfo <- Ginfo[comgene,]

a <- rownames(counts)

b <- rownames(Ginfo)

identical(a,b)

counts$Gene <- as.character(Ginfo$genename)

counts <- counts[!duplicated(counts$Gene),]

rownames(counts) <- counts$Gene

counts <- counts[,-ncol(counts)]

tumor <- colnames(counts)[substr(colnames(counts),14,16) == "01A"]

counts_01A <- counts[,tumor]

library(tidyverse)

if(!require(DESeq2))BiocManager::install('DESeq2')

library(DESeq2)

counts = counts[apply(counts, 1, function(x) sum(x > 1) > 32), ]

conditions=data.frame(sample=colnames(counts),

group=factor(ifelse(substr(colnames(counts),14,16) == "01A","T","N"),levels = c("N","T"))) %>%

column_to_rownames("sample")

dds <- DESeqDataSetFromMatrix(

countData = counts,

colData = conditions,

design = ~ group)

dds <- DESeq(dds)

resultsNames(dds)

res <- results(dds)

save(res,file = "LUAD_DEG.rda")

res_deseq2 <- as.data.frame(res)%>%

arrange(padj) %>%

dplyr::filter(abs(log2FoldChange) > 2, padj < 0.05)

fpkm1 = read.table(file = 'TCGA-LUAD.htseq_fpkm.tsv', sep = '\t', header = TRUE)

rownames(fpkm1) <- fpkm1[,1]

fpkm1 = fpkm1[,-1]

table(substr(colnames(fpkm1),14,16))

fpkm1 <- fpkm1[,substr(colnames(fpkm1),14,16)%in% c("01A","11A")]

table(substr(colnames(fpkm1),14,16))

rownames(fpkm1) <- substr(rownames(fpkm1),1,15)

fpkm <- fpkm1

comgene <- intersect(rownames(fpkm),rownames(Ginfo))

fpkm <- fpkm[comgene,]

Ginfo <- Ginfo[comgene,]

fpkm$Gene <- as.character(Ginfo$genename)

fpkm <- fpkm[!duplicated(fpkm$Gene),]

rownames(fpkm) <- fpkm$Gene

fpkm <- fpkm[,-ncol(fpkm)]

write.table(fpkm, file = "LUAD_fpkm_mRNA_all.txt",sep = "\t",row.names = T,col.names = NA,quote = F)

tumor <- colnames(fpkm)[substr(colnames(fpkm),14,16) == "01A"]

fpkm_01A <- fpkm[,tumor]

normal <- colnames(fpkm)[substr(colnames(fpkm),14,16) == "11A"]

fpkm_11A <- fpkm[,normal]

**Volcano Plot**

install.packages("readxl")

library(readxl)

Ferr <- read_excel("ferroptosis_gene.xlsx")

Ferr=as.data.frame(Ferr)

Ferr = Ferr[,-1]

comgene <- intersect(rownames(res_deseq2),rownames(Ferr))

res_deseq2 <-res_deseq2[comgene,]

class(res_deseq2)

class(Ferr)

Ferr <- Ferr[comgene,]

a <- rownames(res_deseq2)

b <- rownames(Ferr)

identical(a,b)

DEG <- as.data.frame(res)%>%

arrange(padj) %>%

dplyr::filter(abs(log2FoldChange) > 0, padj < 0.05)

logFC_cutoff <- 1

type1 = (DEG$padj < 0.05)&(DEG$log2FoldChange < -logFC_cutoff)

type2 = (DEG$padj < 0.05)&(DEG$log2FoldChange > logFC_cutoff)

DEG$change = ifelse(type1,"DOWN",ifelse(type2,"UP","NOT"))

table(DEG$change)

install.packages("ggpubr")

install.packages("ggthemes")

library(ggpubr)

library(ggthemes)

DEG$logP <- -log10(DEG$padj)

ggscatter(DEG, x = "log2FoldChange", y = "logP",

color = "change",

palette = c("blue", "black", "red"),

size = 1,

label = DEG$Label,

font.label = 8,

repel = T,

xlab = "log2FoldChange",

ylab = "-log10(Adjust P-value)") +

theme_base() +

geom_hline(yintercept = -log10(0.05), linetype = "dashed") +

geom_vline(xintercept = c(-1, 1), linetype = "dashed")

dev.off()

**Correlation Analysis**

gene <- c("AKR1C2")

a <- fpkm_01A[gene,]

b <- fpkm_11A[gene,]

a <- t(a)

b <- t(b)

class(a)

a <- as.data.frame(a)

b <- as.data.frame(b)

a <- a %>% t() %>% as.data.frame()

b <- b %>% t() %>% as.data.frame()

a$disease <- 01

b$disease <- 11

data <- rbind(a,b)

library(ggplot2)

library(ggpubr)

str(data)

data$disease <- as.factor(data$disease)

summary(data)

shapiro.test(data$AKR1C2)

bartlett.test(AKR1C2 ~ disease, data = data)

t_test_result <- t.test(AKR1C2 ~ disease, data = data)

print(t_test_result)

wilcox_test_result <- wilcox.test(AKR1C2 ~ disease, data = data)

print(wilcox_test_result)

ggviolin(data, x = "disease", y = "AKR1C2",

fill = "disease", palette = "npg",

add = c("boxplot", "jitter"),

add.params = list(jitter = 0.2, boxwidth = 0.1)) +

stat_compare_means(method = "wilcox.test",

label = "p.format",

label.x = 1.4) +

theme(plot.title = element_text(face = "bold", size = 14),

axis.text = element_text(color = "black"))

#install.packages("BiocManager")

#if(!require(DESeq2))BiocManager::install('DESeq2')

library(DESeq2)

library(tidyverse)

counts_01A <- read.table("LUAD_counts_mRNA_01A.txt",sep = "\t",row.names = 1,check.names = F,stringsAsFactors = F,header = T)

exp <- read.table("LUAD_fpkm_mRNA_01A.txt", sep = "\t",row.names = 1,check.names = F,header = T)

com <- intersect(colnames(counts_01A),colnames(exp))

exp <- exp[,com]

counts_01A <- counts_01A[,com]

identical(colnames(counts_01A),colnames(exp))

gene <- "AKR1C2"

med=median(as.numeric(exp[gene,]))

conditions=data.frame(sample=colnames(exp),

group=factor(ifelse(exp[gene,]>med,"high","low"),levels = c("low","high"))) %>%

column_to_rownames("sample")

dds <- DESeqDataSetFromMatrix(

countData = counts_01A,

colData = conditions,

design = ~ group)

dds <- DESeq(dds)

resultsNames(dds)

res <- results(dds)

save(res,file="res_deseq2_CDCA3.Rda")

res_deseq2 <- as.data.frame(res)%>%

arrange(padj) %>%

dplyr::filter(abs(log2FoldChange) > 2, padj < 0.05)

**GO**

#install.packages("tidyverse")

#install.packages("BiocManager")

BiocManager::install('clusterProfiler')

#BiocManager::install('org.Hs.eg.db')

library(tidyverse)

library("BiocManager")

library(org.Hs.eg.db)

library(clusterProfiler)

DEG <- as.data.frame(res)%>%

arrange(padj) %>%

dplyr::filter(abs(log2FoldChange) > 2, padj < 0.05)

DEG <- DEG %>% rownames_to_column("Gene")

genelist <- bitr(DEG$Gene, fromType="SYMBOL",

toType="ENTREZID", OrgDb='org.Hs.eg.db')

DEG <- inner_join(DEG,genelist,by=c("Gene"="SYMBOL"))

ego <- enrichGO(gene = DEG$ENTREZID,

OrgDb = org.Hs.eg.db,

ont = "all",

pAdjustMethod = "BH",

minGSSize = 1,

pvalueCutoff =0.05,

qvalueCutoff =0.05,

readable = TRUE)

ego_res <- ego@result

save(ego,ego_res,file = "GO_CDCA3_DEG.Rdata")

barplot(ego, showCategory = 20,color = "pvalue")

barplot(ego, drop = TRUE, showCategory =10,split="ONTOLOGY") +

facet_grid(ONTOLOGY~., scale='free')

**KEGG**

#install.packages("tidyverse")

#install.packages("BiocManager")

BiocManager::install('clusterProfiler')

#BiocManager::install('org.Hs.eg.db')

library(tidyverse)

library("BiocManager")

library(org.Hs.eg.db)

library(clusterProfiler)

DEG <- as.data.frame(res)%>%

arrange(padj) %>%

dplyr::filter(abs(log2FoldChange) > 1, padj < 0.05)

DEG <- DEG %>% rownames_to_column("Gene")

genelist <- bitr(DEG$Gene, fromType="SYMBOL",

toType="ENTREZID", OrgDb='org.Hs.eg.db')

DEG <- inner_join(DEG,genelist,by=c("Gene"="SYMBOL"))

kk <- enrichKEGG(gene = DEG$ENTREZID,

organism = 'hsa',

pvalueCutoff = 0.6,

qvalueCutoff =0.6)

kk_res <- kk@result

save(kk,kk_res,file = "KEGG_CDCA3_DEG.Rdata")

kk <- setReadable(kk, OrgDb = org.Hs.eg.db, keyType="ENTREZID")#基因数字变名称

load("KEGG_CDCA3_DEG.Rdata")

barplot(kk, showCategory = 20,color = "pvalue")+

scale_y_discrete(labels=function(x) str_wrap(x, width = 100))

dev.off()

**GSEA**

#install.packages("tidyverse")

#install.packages("BiocManager")

BiocManager::install('clusterProfiler')

#BiocManager::install('org.Hs.eg.db')

library(tidyverse)

library("BiocManager")

library(org.Hs.eg.db)

library(clusterProfiler)

DEG <- as.data.frame(res)%>%

arrange(padj) %>%

dplyr::filter(abs(log2FoldChange) > 1, padj < 0.05)

DEG <- DEG %>% rownames_to_column("Gene")

genelist <- bitr(DEG$Gene, fromType="SYMBOL",

toType="ENTREZID", OrgDb='org.Hs.eg.db')

DEG <- inner_join(DEG,genelist,by=c("Gene"="SYMBOL"))

hall_GMTs <- "h.all.v2023.1.Hs"

Hallmarker <- "c5.all.v2023.1.Hs.entrez.gmt"

kegmt <- read.gmt(file.path(hall_GMTs,Hallmarker))

geneList = DEG[,3]

names(geneList) = as.character(DEG[,'ENTREZID'])

head(geneList)

geneList = sort(geneList, decreasing = TRUE)

set.seed(1)

KEGG<-GSEA(geneList,TERM2GENE = kegmt)

KEGG_result_df <- as.data.frame(KEGG)

write.table(KEGG_result_df,file="GSEA_hall_result.txt",sep = "\t",row.names = T,col.names = NA,quote = F)

save(KEGG,KEGG_result_df,file = "GSEA_deg_AKR1C2.rda")

library(enrichplot)

gseaplot2(KEGG, geneSetID = 1:10, subplots = 1:3),base_size = 12,rel_heights = c(1.5, 0.5, 1),subplots = 1:3,pvalue_table = T)

dev.off()

**ROC and Correlation Analysis**

clincal = read.table(file = 'TCGA-LUAD.clinical.tsv', sep = '\t', header = TRUE)

survival = read.table(file = 'TCGA-LUAD.survival.tsv', sep = '\t', header = TRUE)

write.table(clincal,"clincal.txt",sep = "\t",row.names = T,col.names = NA,quote = F)

write.csv(clincal, file = "clincal.csv")

write.table(survival,"survival.txt",sep = "\t",row.names = T,col.names = NA,quote = F)

write.csv(survival, file = "survival.csv")

fpkm_01A <- read.table("LUAD_fpkm_mRNA_01A.txt",sep = "\t",row.names = 1,check.names = F,stringsAsFactors = F,header = T)

fpkm_11A <- read.table("LUAD_fpkm_mRNA_11A.txt",sep = "\t",row.names = 1,check.names = F,stringsAsFactors = F,header = T)

gene <- c("AKR1C2")

a <- fpkm_01A[gene,]

b <- fpkm_11A[gene,]

a <- t(a)

b <- t(b)

class(a)

a <- as.data.frame(a)

b <- as.data.frame(b)

a <- a %>% t() %>% as.data.frame()

b <- b %>% t() %>% as.data.frame()

write.csv(a, file = "01A-AKR1C2.csv")

write.csv(b, file = "11A-AKR1C2.csv")

clinical <- data.table::fread("clincal.csv",data.table = F)

rownames(clinical) <- clinical[,1] #Alt <-

clinical = clinical[,-1]

comgene <- intersect(rownames(clinical),rownames(a))

clinical <- clinical[comgene,]

a <- a[comgene,]

a <- as.data.frame(a)

clinical$AKR1C2 <- as.character(a$a)

write.csv(clinical, file = "AKR1C2_clinical.csv")

head(clincal)

rownames(clincal) <- clincal[,1]

clincal = clincal[,-1]

rownames(clincal) <- gsub("-",".",rownames(clincal))

comgene <- intersect(rownames(clinical),rownames(clincal))

clincal <- clincal[comgene,]

c<- rownames(clincal)

d<- rownames(clinical)

identical(c,d)

clinical$OS.time <- as.character(clincal$days_to_death.demographic)

write.csv(clinical, file = "AKR1C2_clinical_2.csv")

rownames(survival) <- survival[,1]

survival = survival[,-1]

AKR <- data.table::fread("01A-AKR1C2.csv",data.table = F)

comgene <- intersect(rownames(clinical),rownames(clincal))

rownames(survival) <- gsub("-",".",rownames(survival))

comgene <- intersect(rownames(survival),AKR$V1)

survival1 <- survival[comgene,]

AKR1 <- AKR

rownames(AKR1) <- AKR1[,1]

AKR1 <- AKR1[comgene,]

c<- rownames(AKR1)

d<- rownames(survival1)

identical(c,d)

survival1$AKR1C2 <- as.character(AKR1$AKR1C2)

gene <- "AKR1C2"

med=median(as.numeric(survival1$AKR1C2))

survival1$group <- ifelse(survival1$AKR1C2>med,"High","Low")

survival1$OS.time <- survival1$OS.time*12

survival1$group <- factor(survival1$group, levels = c("Low","High"))

class(survival1$group)

table(survival1$group)

#install.packages("survival")

library(survival)

fitd <- survdiff(Surv(OS.time, OS) ~ group,

data = survival1,

na.action = na.exclude)

pValue <- 1 - pchisq(fitd$chisq, length(fitd$n) - 1)

fit <- survfit(Surv(OS.time, OS)~ group, data = surv)

summary(fit)

plot(fit, conf.int = T,

col = c("blue", "red"),

lwd = 2,

xlab = "Time(Months)",

ylab = "Survival probablity(%)"

)

legend("topright",

title = "Group",

c("Low", "High"),

lwd = 2, lty = 1,

col = c("blue", "red"))

p.lab <- paste0("P", ifelse(pValue < 0.001, " < 0.001", paste0(" = ",round(pValue, 3))))

text(25, 0.2, p.lab)

dev.off()

library(ggplot2)

library(ggpubr)

str(clinical)

clinical$Gender <- as.factor(clinical$Gender)

summary(clinical)

shapiro.test(clinical$AKR1C2)

bartlett.test(AKR1C2 ~ Gender, data = clinical)

t_test_result <- t.test(AKR1C2 ~ Gender, data = clinical)

print(t_test_result)

wilcox_test_result <- wilcox.test(AKR1C2 ~ Gender, data = clinical)

print(wilcox_test_result)

ggviolin(clinical, x = "Gender", y = "AKR1C2",

fill = "Gender", palette = "npg",

add = c("boxplot", "jitter"),

add.params = list(jitter = 0.2, boxwidth = 0.1)) +

stat_compare_means(method = "wilcox.test",

label = "p.format",

label.x = 1.4) +

theme(plot.title = element_text(face = "bold", size = 14),

axis.text = element_text(color = "black"))
